# Nationally representative SARS-CoV-2 antibody prevalence estimates after the first epidemic wave in Mexico

**DOI:** 10.1038/s41467-022-28232-9

**Published:** 2022-02-01

**Authors:** Ana Basto-Abreu, Martha Carnalla, Leticia Torres-Ibarra, Martín Romero-Martínez, Jesús Martínez-Barnetche, Irma López-Martínez, Rodrigo Aparicio-Antonio, Teresa Shamah-Levy, Celia Alpuche-Aranda, Juan A. Rivera, Tonatiuh Barrientos-Gutierrez, ENSANUT-COVID collaborators, Lucia Cuevas-Nasu, Elsa Berenice Gaona-Pineda, Marco Antonio Ávila-Arcos, Francisco Reyes-Sánchez, Rossana Torres-Álvarez, Nancy López-Olmedo, Desiree Vidaña-Perez, Romina González-Morales, David Barrera-Nuñez, Carolina Perez-Ferrer, Carlos Gaspar-Castillo, Dalia Stern, Francisco Canto-Osorio, Andrés Sanchez-Pájaro

**Affiliations:** 1 Center for Population Health Research, National Institute of Public Health, Cuernavaca, Mexico; 2 Center for Research in Evaluation and Surveys, National Institute of Public Health, Cuernavaca, Mexico; 3 Center for Research on Infectious Diseases, National Institute of Public Health, Cuernavaca, Mexico; 4 Institute for Epidemiological Diagnosis and Reference, Mexico City, Mexico; 5 National Institute of Public Health, Cuernavaca, Mexico; 6 CONACYT- National Institute of Public Health, Cuernavaca, Mexico

**Keywords:** Infectious diseases, Epidemiology, Outcomes research, SARS-CoV-2

## Abstract

Seroprevalence surveys provide estimates of the extent of SARS-CoV-2 infections in the population, regardless of disease severity and test availability. In Mexico in 2020, COVID-19 cases reached a maximum in July and December. We aimed to estimate the national and regional seroprevalence of SARS-CoV-2 antibodies across demographic and socioeconomic groups in Mexico after the first wave, from August to November 2020. We used nationally representative survey data including 9,640 blood samples. Seroprevalence was estimated by socioeconomic and demographic characteristics, adjusting by the sensitivity and specificity of the immunoassay test. The national seroprevalence of SARS-CoV-2 antibodies was 24.9% (95%CI 22.2, 26.7), being lower for adults 60 years and older. We found higher seroprevalence among urban and metropolitan areas, low socioeconomic status, low education and workers. Among seropositive people, 67.3% were asymptomatic. Social distancing, lockdown measures and vaccination programs need to consider that vulnerable groups are more exposed to the virus and unable to comply with lockdown measures.

## Abstract (web summary)

SARS-CoV-2 seroprevalence surveys provide estimates of the extent of prior infection in a population. In this nationally representative survey from Mexico, the authors estimate seroprevalence after the first epidemic wave at ~25%, with variation by region, age, socioeconomic status, and education level.

## Introduction

Nationally representative SARS-CoV-2 seroprevalence surveys estimate the extent of infection in the population independently of severity and test availability^1^. In 2020, SARS-CoV-2 seroprevalence studies were conducted in various countries using representative country-level data. In the first COVID-19 wave (before June 2020), seroprevalence ranged from 1.4% in urban areas in Brazil to 17.1% in Iran (see Supplementary Fig. 7)^2,3^. Between July and August 2020, a study in the US found that seroprevalence ranged from <1% to 23%^4^. Between October and November 2020, a study in Colombia found seroprevalence ranged from 27% in Medellin to 59% in Leticia^5^. In Mexico, seroprevalence studies have been limited to specific groups: 5.7% among governmental workers in Guadalupe City and 29.5% among ambulatory patients in a private laboratory in Veracruz^6,7^. Yet, to date no nationally representative estimates have been provided.

As other Latin American countries, Mexico is subject to deep social inequalities that translate into different odds of adhering to mitigation recommendations, such as lockdowns^8^. Using surveillance data, prior studies in Mexico have suggested that COVID-19 infections could be higher among low socioeconomic groups^9^. Yet, surveillance data is limited by healthcare access and overrepresents symptomatic and severe cases of COVID-19, clouding the real magnitude of infection across socioeconomic groups^10^.

In 2020 Mexico experienced two waves of COVID-19 that reached their peak in July and December. This pattern was similar across regions, although some areas, like Mexico City, maintained a high level of transmission. In response to the emergency, a nationally representative serosurvey was implemented between August and November just after the first wave subsided. Using data from this survey, we aimed to estimate the seroprevalence of SARS-CoV-2 antibodies in Mexico at the national and regional level. We also explored the demographic and socioeconomic characteristics associated with seropositivity.

## Results

**Fig. 1:**
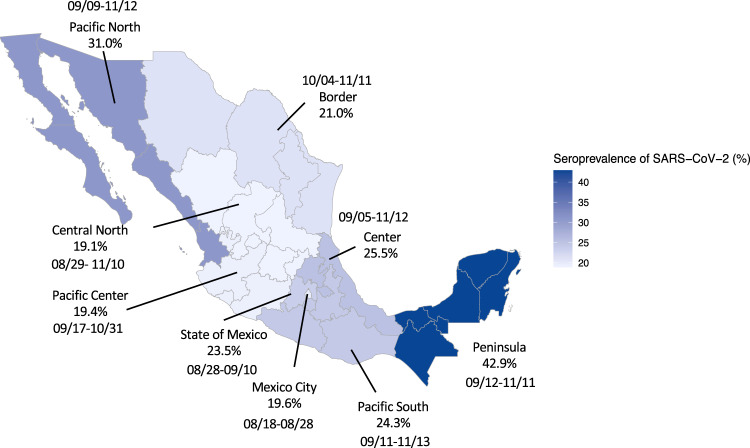
Seroprevalence of SARS-CoV-2 across nine regions  in Mexico.

Figure 1 presents the seroprevalence of SARS-CoV-2 in the nine Mexican regions between August and November 2020. The seroprevalence in Mexico in the period of August to November 2020 was 24.9%. The regions with the highest seroprevalence were Pacific North (31.0%) and Peninsula (42.9%) and the regions with the lowest were Central North (19.1%) and Pacific Center (19.4%).

Prevalence for each region is presented as percentage. Light blue color regions had the lowest seroprevalence and dark blue the highest. The collection period was from August to November 2020, and varied by region as shown (starting month/day-ending month/day).

Supplementary Table 1 shows the characteristics of the study population, representing 125 million inhabitants. By age, 33.0% were children and adolescents, 30.9% were adults less than 40 years, 23.3% were adults 40–59, and 12.8% were adults 60 and older. Also, 37.7% of individuals had elementary school or less, 25.0% middle school, 19.8% high school, and 17.5% graduate school. Table 1 presents the seroprevalence of SARS-CoV-2 by sociodemographic characteristics. The highest seroprevalence of SARS-CoV-2 was estimated among adults 20-39 years (27.9%) and 40–59 years (27.8%), compared with adults 60 and older (18.6%). Rural areas had a 21.1% seroprevalence (95%CI 16.8, 25.4), in comparison with 27.1% (95%CI 23.4, 30.9) in urban and 25.2% (95%CI 22.7, 27.8) in metropolitan areas. Formal (30.1%; 95%CI 26.1, 33.3) and informal workers (28.0%; 95%CI 24.7,30.6) had the highest anti-SARS-CoV-2 antibodies, as compared to students (22.3%; 95CI 16.5, 26.9), unemployed (24.0%; 95%CI 20.9, 26.3) and retired participants (16.5%; 95%CI 11.7, 20.7).

**Table 1:** Seroprevalence of antibodies anti-SARS-CoV-2 by sociodemographic characteristics. Mexico 2020.

	Seroprevalence % (95%CI)
National	24.9 (22.2, 26.7)
*Age (years)*	
1–19	22.5 (19.0, 25.3)
20–39	27.9 (24.9, 30.3)
40–59	27.8 (24.6, 30.2)
60 and older	18.6 (15.4, 20.8)
*Sex*	
Male	25.3 (22.3, 27.4)
Female	24.5 (21.7, 26.5)
*Education*	
Elementary school or less	22.2 (19.1, 24.3)
Middle school	28.3 (24.6, 31.3)
High school	27.5 (23.9, 30.3)
Graduate	22.4 (18.9, 25.1)
*Employment status*^a^	
Unemployed	24.0 (20.9, 26.3)
Student	22.3 (16.5, 26.9)
Retired	16.7 (11.7, 20.7)
Formal worker^b^	30.1 (26.1, 33.3)
Informal worker	28.0 (24.7, 30.6)
Socioeconomic level	
Low	27.8 (24.2, 31.3)
Medium	24.6 (21.8, 27.4)
High	22.3 (19.5, 25.1)
Urbanization	
Rural	21.1 (16.8, 25.4)
Urban	27.1 (23.4, 30.9)
Metropolitan	25.2 (22.7, 27.8)

*CI* confidence interval.

^a^ 15 years of age and more.

^b^ Worker with access to social security services or private medical insurance.

Table 2 shows the sociodemographic factors associated with SARS-CoV-2 seroprevalence in Mexico in 2020. For children and adolescents none of the variables included were associated with seropositivity. Adults aged 60–69 years presented 33% lower seropositivity and those of 70 and older presented 46% lower seropositivity in comparison to participants aged 20–29 years. Lower education was associated with higher seropositivity. Participants living in urban and metropolitan areas had 34% and 46% higher seropositivity than those living in rural areas. Formal workers had 29% higher seropositivity than participants without employment. Finally, individuals in the low socioeconomic status (SES) group had 20% higher seropositivity than participants in the high SES group.

**Table 2:** Sociodemographic factors associated with seropositivity to antibodies anti SARS-CoV-2 in Mexico, 2020. Multivariable Poisson regression models^a^.

	Children and adolescents		Adults	
	PR % (95% CI)	*p-*value	PR % (95% CI)	*p-*value
*Age group (years)*				
1–9	REF			
10–19	1.21 (0.97, 1.51)	0.097		
20–29			REF	
30–39			0.94 (0.81, 1.09)	0.421
40–49			0.99 (0.85, 1.15)	0.873
50–59			0.87 (0.73, 1.03)	0.103
60–69			0.77 (0.62, 0.96)	0.018
70+			0.54 (0.42, 0.70)	<0.001
*Sex*				
Male	REF		REF	
Female	1.01 (0.81, 1.26)	0.956	1.00 (0.90, 1.10)	0.946
*Education*				
Graduate/postgraduate			REF	
High school			1.23 (1.04, 1.45)	0.017
Middle school			1.32 (1.11, 1.57)	0.002
Elementary school or less			1.30 (1.08, 1.57)	0.006
*Urbanization*				
Rural	REF		REF	
Urban	1.42 (0.96, 2.10)	0.077	1.34 (1.09, 1.66)	0.006
Metropolitan	1.43 (0.94, 2.18)	0.091	1.46 (1.18, 1.81)	0.001
*Employment status*				
Unemployed			REF	
Student			0.90 (0.64, 1.27)	0.549
Retired			1.00 (0.75, 1.34)	0.997
Formal worker^b^			1.29 (1.11, 1.51)	0.001
Informal worker			1.13 (0.99, 1.28)	0.066
*Socioeconomic level*				
High	REF		REF	
Medium	0.93 (0.67, 1.29)	0.646	1.11 (0.96, 1.29)	0.147
Low	1.15 (0.78, 1.69)	0.484	1.20 (1.01, 1.42)	0.037

*PR* prevalence ratio, *REF* reference group, *CI* confidence interval.

^a^Both multivariable regression models were adjusted by region and the covariates listed in the table. A two-sided F test was used to evaluate the overall model and a two-sided T test for each variable in the model. Degrees of freedom=6.

^b^Worker with access to social security services or private medical insurance.

Overall, 67.3% of seropositive participants were asymptomatic, 21.5% symptomatic and 11.2% pauci-symptomatic. The proportion of asymptomatic participants was similar by sex, but varied by age group: 82.4% among children and adolescents, compared to 63.9% among adults 20–39, 56.7% among adults 40–59, and 60.6% among adults 60 and older.

Table 3 shows the results of the sensitivity analysis. The national seroprevalence was estimated to be 24.9% (95%CI 22.2, 26.7) adjusting for the in-house test performance, in comparison to 23.2% (95%CI 21.0, 25.3) using the manufacturer’s test performance and to 23.8%, (95%CI 21.3, 25.4) using the selection bias adjustment, with small variations across regions; the overall patterns remained unchanged.

**Table 3:** Sensitivity analysis for the seroprevalence of antibodies SARS-CoV-2 in Mexico, by regions. Ensanut 2020 COVID-19.

	Prevalence adjusted by in-house test performance (main scenario)		Prevalence adjusted by manufacturer test performance		Prevalence adjusted by selection bias^a^	
	%	95%CI	%	95%CI	%	95%CI
National	24.9	22.2, 26.7	23.2	21.0, 25.3	23.8	21.3, 25.4
Region						
Pacific North	31.0	25.0, 36.6	28.8	23.2, 34.4	30.2	24.1, 35.6
North Border	21.0	15.6, 25.5	19.5	14.9, 24.0	20.3	15.0, 24.8
Pacific Center	19.4	12.7, 25.2	18.1	12.7, 23.9	18.3	11.9, 23.5
Center North	19.1	14.6, 22.6	17.7	14.0, 21.5	17.8	13.1, 21.5
Center	25.5	20.7, 29.6	23.7	19.4, 27.9	24.3	19.1, 28.7
Mexico City	19.6	14.7, 23.8	18.2	14.0, 22.3	18.3	13.6, 22.1
State of Mexico	23.5	18.3, 27.7	21.8	17.4, 26.3	22.2	17.1, 26.4
Pacific South	24.3	17.2, 30.2	22.7	16.9, 28.4	23.6	17.3, 29.2
Peninsula	42.9	36.3, 49.0	39.6	33.1, 45.7	41.5	34.8, 47.9

*CI* confidence interval.

^a^Adjusted for selection bias considered symptom distribution in the household questionnaire. Adjusted by in-house test performance.

## Discussion

We aimed to estimate the prevalence of SARS-CoV-2 antibodies in Mexico using a nationally representative sample. The estimated national prevalence of SARS-CoV-2 antibodies in Mexico between August and November 2020 was 24.9%. The highest seroprevalence was observed in the Peninsula region (42.9%), and the lowest in the Central North region (19.1%). Seroprevalence was higher among workers, low socioeconomic groups, low education and urban and metropolitan areas among adults. We also found that 67% of the seropositive cases were asymptomatic.

The national seroprevalence in Mexico was similar to levels observed in cities in Colombia and some states in the US, collected in similar periods. Most seroprevalence studies were conducted during the first wave (April and May, 2020) and reported seroprevalences between 5% and 15% (see Supplementary Fig. 7)^2,3,11–14^. Globally, few seroprevalence studies were collected in the second half of 2020. Between August and November 2020, we found seroprevalences that ranged from 4.3% in Florida to 59% in Leticia, Colombia^4,5^. The seroprevalence in Mexico City (19.6%) and in the State of Mexico (23.5%) was similar to the one observed in New York (17–23.3%) between August and September^4^. Other Mexican regions presented similar seroprevalences to Medellin (27%) and Bogotá (30%), but lower than Leticia (59%) and Barranquilla (55%) in Colombia^5^.

Mexico SARS-CoV-2 seroprevalence was heterogenous by region and time. The highest seroprevalence in Mexico was observed in the Peninsula up to November 2020 (42.9%), when the surveillance system indicated a cumulative incidence of 586/100,000 inhabitants. In contrast, Mexico City had a 19.6% seroprevalence in August, when the surveillance system estimated a cumulative incidence of 1,067/100,000 inhabitants (see Supplementary Table 12). These results suggest that the intensity of testing was heterogeneous across regions; for instance, Mexico City performed 5,931 tests/100,000 inhabitants until September 30th (midpoint of the survey), compared to 1,377 tests/100,000 inhabitants in the Peninsula. These results are consistent with a systematic review, which found heterogeneous rates of incident cases compared to seroprevalence^15^. This finding highlights the importance of seroprevalence surveys, which can supersede the limitations of surveillance systems and provide better estimates of the impact of the SARS-CoV-2 pandemic in the population.

We found that the working population, people in the lower socioeconomic status or lower education had higher seroprevalence levels among adults than their counterparts. Differences in seropositivity suggest that the COVID-19 pandemic affected people differentially, with higher infection rates among vulnerable groups. People from disadvantaged socio-economic groups in Mexico tend to work in essential activities, like food provision and transportation, but they also have strong participation in the informal economic sector and could have had lower chances to comply with stay-at-home directives^16^. Also, low socioeconomic groups in Mexico may be more susceptible to household transmission, because they tend to live in crowded and multigenerational settings^17^. In Brazil, people in the poorest quantile of wealth presented 43% higher odds of being seropositive, compared with the richest quantile^3^. In Cape Town, South Africa, seropositivity was associated with informal housing, living in low-income districts, and low-wage^18^. In Israel, seropositivity was 6.5% in the low socioeconomic status compared to 1.6% in high socioeconomic status and 4.3% in large municipalities compared to 3.4% in small municipalities^19^. In Lima, Peru, participants in the low socioeconomic status were 3.4 times more likely to be seropositive than participants in the high socioeconomic group^20^. Considering that COVID-19 is transforming families’ day-to-day lives, with short and middle-term needs for health-care and rehabilitation and even permanent sequelae, address the socioeconomic gap of COVID-19 is urgent. Efforts must be made to facilitate compliance with mitigation measures, but also, economic policies need to be put in place to help low socioeconomic people fare better with COVID-19.

Asymptomatic cases are not regularly tested in the Mexican surveillance system; as a consequence, before this study we had no information about the proportion of the population positive to SARS-CoV-2 that experienced no symptoms. We observed a high proportion of asymptomatic persons, 67%, compared to Austria (20%), UK (20%), Spain (36%), and Iran (36%), but similar to Mexican governmental workers (59%), and people in Lima-Peru (56%) and lower than in China (82%)^2,6,14,21–23^. The large proportion of asymptomatic cases could be related to recall bias (considering that several months have passed since infection for some cases), disregard for mild and common symptoms, such as fatigue or headache, and bias due to report-by-proxy since symptoms for all household members were reported by the head of the household to reduce collection times.

The present study has some limitations to be discussed. Data were collected in different time frames thus, seroprevalence across regions should be interpreted cautiously. As the serologic survey had a low response rate, we conducted a sensitivity analysis to evaluate potential sources of bias. We found that individuals with COVID-19 related symptoms were more likely to participate in the serologic sample, biasing the seroprevalence estimate upwards. After bias quantification, the seroprevalence at the national level was 23.8%, (95%CI 21.3, 25.4), although the confidence intervals overlapped with the original estimate 24.9% (95%CI 22.2, 26.7). False positives have been reported in areas where malaria is endemic^24^, thus, we used our in-house validation with a random sample of all states in Mexico, but we only observed one false positive; thus, cross-reactivity did not seem to be a problem in Mexico.

In Mexico, the ongoing pandemic affected at least a quarter of the population by the end of November, 2020. Differences in seroprevalence suggest that workers, people with low educational level and living in urban and metropolitan areas were more frequently exposed to the virus. Mexico needs to address structural vulnerabilities and use this opportunity to rethink their public policies grounded on equality. This includes providing healthcare for infected people, food support, preserving jobs, and compensate for salaries’ reduction due to lockdown measures^16,25^. Besides, implementing participatory interventions and designing evidence-based vaccination plans is key to protect the most vulnerable population. One year after the pandemic, we have the chance to address inequity and avoid widening the health gap that already existed in Mexico.

## Methods

### Study design

The 2020 National Health and Nutrition Survey (Ensanut) focused in understanding the effects of the pandemic on health, food security, dietary quality, and access to healthcare services in Mexico. The survey was conducted from August to November 2020 and used a probabilistic, multistage, stratified, and clustered sampling strategy to be representative of the national, regional, and rural/urban levels. Using this sampling strategy, 10,216 households were selected. At each household, an adult family member was asked to respond to a household questionnaire and a questionnaire on the use of health services and health status of each family member. All research procedures were approved by the ethics, research and biosafety boards from the National Institute of Public Health. The information in the field was collected on tablets through a capture system developed in the CSPro language version 7.5.0. Further details about the sampling strategy and external validity are available in Supplementary methods (section 2).

**Fig. 2:**
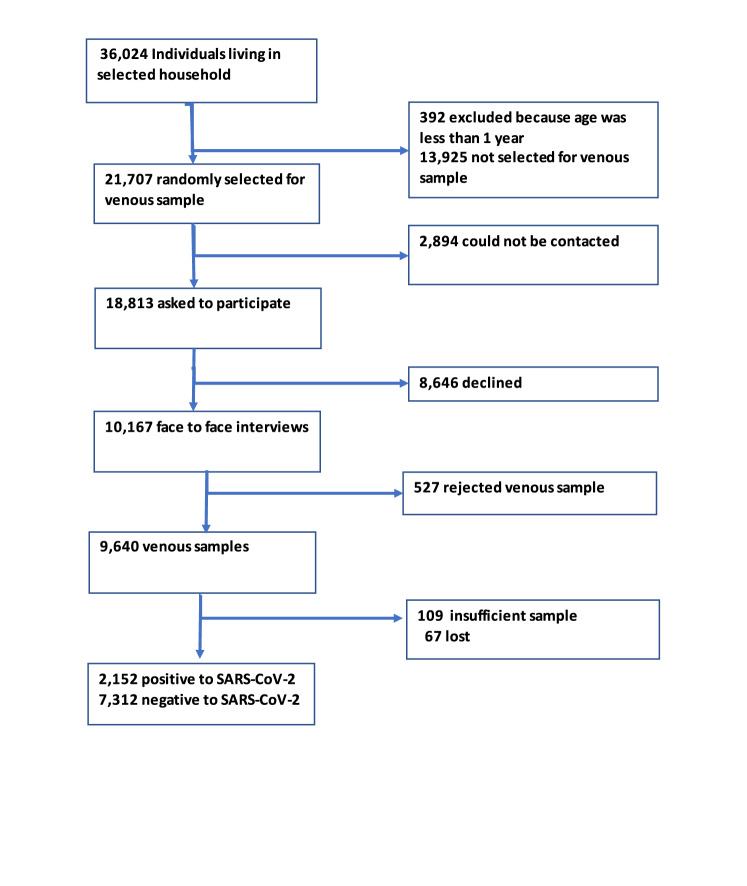
Flowchart of participants in the serologic subsample in Ensanut 2020. The flowchart of participants in the National Health and Nutrition Survey (Ensanut 2020). From the 36,024 subjects selected at the household level, a subsample of 21,707 were randomly selected to participate in the serologic sample. From those some could not be contacted and others declined to participate. We collected 9,640 venous samples.

From 35,632 eligible participants in the household survey, 21,707 individuals were randomly selected to provide a blood sample following a multistage and stratified selection strategy divided into six age groups: 1–4 years, 5–9 years, 10–19 years, 20–34 years, 35–49 years and 50+ years. From those, 2894 could not be contacted. A total of 9640 blood samples were collected, for a 51% response rate from contacted participants and 44% from eligible participants. From the 21,707 eligible individuals, 13% could not be contacted, 40% declined and 2% rejected being punctured (Fig. 2).

### Determination of antibodies

Ensanut 2020 COVID-19 considered the determination of immunoglobulin G against nucleocapsid (N) and spike (S) proteins. This paper considers the seroprevalence of antibodies to the N protein, since S protein is still under analysis. Blood samples were centrifuged in the field to separate the serum and were frozen to be delivered to the National Institute of Public Health. Samples were then sent to the Institute for Epidemiological Diagnosis and Reference for analysis using the Roche Elecsys Anti-SARS-CoV-2pan-immunoglobulin immunoassay test (Ref 09203095190, Roche, Switzerland). According to the manufacturer’s recommendations, samples were considered reactive using a threshold of ≥1.0 AU/ml. We validated the test using pre-COVID-19 serum samples as controls and serum samples from people with confirmed COVID-19 by RT-PCR that were obtained at least 22 days after symptoms onset, when the sensitivity of the antibody tests is the highest. The validation test showed a sensitivity of 92.02% (95% CI 88.57–94.50) and a specificity of 99.52% (95% CI 97.35 to 99.92). Further details about the in-house validation are available in Supplementary methods (section 3).

### COVID-19 related symptoms

We constructed a variable of COVID-19 related symptoms to categorize participants in symptomatic, asymptomatic and pauci-symptomatic. The following question was answered by the head of the household in relation to their experience or that of their family members: *“Between March 2020 and today did you (or any family member) present any of the following symptoms?”* Informants were presented with 14 options: cough, fever, headache, sore or burning throat, runny nose, red eyes, muscle or joint pain, difficulty breathing, shortness of air, chest pain, vomiting, diarrhea, loss of smell, or taste, and other symptoms. Supplementary Fig. 8 shows the prevalence of symptoms by serostatus. Participants were classified as symptomatic if they met the “suspected case” definition of COVID-19 of Mexico’s Health Ministry: having at least one major symptom (cough, fever, headache, shortness of breath, or air in the lungs or chest pain) and one minor symptom (sore or burning throat, runny nose, red eyes, pain in muscles or joints, chest pain, loss of smell and loss of taste)^26^. Participants that did not fulfill the definition but experienced at least one symptom were considered “pauci-symptomatic”^14^. Participants with no symptoms were considered asymptomatic.

### Covariates

Sex, age groups (1 to 19, 20-39, 40-59 and 60 years or older), geographic region and urbanization were used as main demographic characteristics. Participants were classified into nine geographic regions: Mexico City, North Border (Chihuahua, Coahuila, Nuevo León, Tamaulipas), Central-Pacific (Colima, Jalisco, Michoacán), Central-North (Aguascalientes, Durango, Guanajuato, Querétaro, San Luis Potosí, Zacatecas), Center (Hidalgo, Tlaxcala, Veracruz), Pacific-North (Baja California, Baja California Sur, Nayarit, Sinaloa, Sonora), State of Mexico, Pacific-South (Guerrero, Morelos, Oaxaca, Puebla), and Peninsula (Campeche, Chiapas, Quintana Roo, Tabasco, Yucatán). Urbanization was divided into rural (<2,500 inhabitants), urban (2,500 to 100,000 inhabitants) or metropolitan (>100,000 inhabitants).

Education was categorized according to the maximum level of completed studies into elementary school, middle school, high school, graduate, or postgraduate. Employment status was constructed for individuals 15 years and older based on the question: “During the past week, did you work at least one hour?”. Those who answered “yes”, were considered employees and further divided into formal, if they had social security healthcare access or informal if they did not. Those who answered “no” were then asked about their activities in the week prior to the survey; based on their response they were classified as “students” or “retirees”, while those who answered “looked for a job”, “have a permanent disability that does not allow me to work”, or “unpaid domestic work” were considered unemployed. A socioeconomic status index was constructed using household’ characteristics (construction materials of the floor, walls, and ceiling, number of bedrooms, running water), own a car, number of household assets (refrigerator, washing machine, microwave, stove, and boiler) and number of electrical devices (tv, cable, radio, telephone, and computer). The index was constructed using Principal Component Analysis, with a polychoric correlation matrix. The first component explaining 50.1% of the total variability was selected with an eigenvalue of 4.0 and categorized into tertiles (low, medium, high).

### Statistical analysis

The observed seroprevalence was calculated as:

1 $${Observed}\,{seroprevalence}=\,\frac{{Number}\,{of}\,{reactive}\,{specimens}}{{Total}\,{specimens}\,{tested}}\,$$

Considering that the Elecsys test is imperfect, with 92.02% of sensitivity and 99.52% of specificity, we adjusted the seroprevalence as follows:^27^

2 $${Adjusted}\,{prevalence}=\,\frac{{Observed}\,{prevalence}\,+\,{Specificity}-1}{{Sensitivity}+{Specificity}-1}$$

To calculate confidence intervals, we simulated values of the observed prevalence using a normal distribution using as parameters the confidence intervals of the observed prevalence estimated from the survey and the confidence intervals of sensitivity and specificity estimated in supplementary methods (section 3). The process was repeated 1,000 times and 95% CIs were calculated from 2.5th and 97.5th quantiles of the bootstrap distribution. We reported the final adjusted seroprevalence estimate as the mean and the 95% uncertainty interval of the bootstrap distribution.

We used sampling weights to adjust the seroprevalence for the selection probabilities and non-response rates to the serologic subsample. Weights were calculated as the inverse probability of selection, adjusted by non-response by post-stratifying the sample on region, sex, age group (<10, 10-19, 20−34, 35−49, ≥50 years), so that the weighted sum of respondents in each stratum matched the total population estimated at the end 2020 by the National Population Council. Additionally, weights were adjusted to resemble the distribution of the reported chronic diseases in the household questionnaire. To estimate adjusted prevalence ratios we used Poisson regression models with robust variance^28^. We fitted a multivariate model to estimate the sociodemographic factors associated with seroposivity stratified by age group (adolescents and children, adults 20-59 years, adults 60 years and older). All analyses considered survey weights using the module “svy” from Stata 14.0 (College Station, TX).

### Sensitivity analysis

#### Information bias

We performed a sensitivity analysis using test performance as reported by the manufacturer (sensitivity 100%, specificity 99.8%)^29^, to compare results to the adjustment made using our in-house validation.

#### Selection bias

We performed a sensitivity analysis to assess the potential impact of specific variables that could inform selection bias, considering a low response rate in the serologic subsample. We analyzed differences in socioeconomic characteristics between participants in the household questionnaire (*n* = 36,024) and in the serologic subsample with valid results (*n* = 9464). We selected variables that could have been associated with seropositivity and with participation in the provision of a blood sample: age, sex, region, education, employment, having reported contact with a suspected case, having experienced a respiratory disease, and having experienced COVID-19 related symptoms. We used *raking*, a sampling balance method^30^, to replicate the distribution of key variables from the household questionnaire into the serologic sample. We used “symptoms” by region and age group (<20, 20–39, 40–59, and 60 and older) as the key variable on raking. After raking the distribution of variables was comparable between the household survey and the serologic sample, with the exception of the “students” category in the education variable (see supplementary Table 11). A detailed explanation of the selection bias quantification procedure is available in supplementary methods (section 4).

### Reporting summary

Further information on research design is available in the Nature Research Reporting Summary linked to this article.

## Supplementary information

Supplementary Information

Reporting Summary

## Data Availability

Data is publicly available in: https://ensanut.insp.mx/encuestas/ensanutcontinua2020/descargas.php; folio_int is the unique identifier and ponde_g20 is the weight variable to expand the results to the Mexican population.

## Code availability

Two scripts in Stata 14.0 are available, one for the main analysis and another for the sensitivity analysis. Also, an excel file is provided to adjust the estimation by in-house test performance and the dataset for the sensitivity analysis are provided in GitHub^31^.
