# Supplementary material for: Nationally representative SARS-CoV-2 antibody prevalence estimates after the first epidemic wave in Mexico
Source: Nat Commun. 2022 Feb 1;13:589. doi: 10.1038/s41467-022-28232-9 (PMC8807586; doi:10.1038/s41467-022-28232-9)
Supplement: Supplementary file 1 — Supplementary Information [file 41467_2022_28232_MOESM1_ESM.pdf]

Supplementary information

Nationally representative SARS-CoV-2 antibody prevalence estimates after the first epidemic wave in Mexico

Ana Basto-Abreu, Martha Carnalla, Leticia Torres-Ibarra, Martín Romero-Martínez, Jesús Martínez-Barnetche, Irma López-Martínez, Rodrigo Aparicio-Antonio, Teresa Shamah-Levy, Celia Alpuche-Aranda. Juan A. Rivera, Tonatiuh Barrientos-Gutierrez, ENSANUT-COVID collaborators.

Contents

Supplementary methods **.......... 2**

1. Study population included in Ensanut COVID 2020 2

2. External validity of ENSANUT COVID-19, 2020 3

2.1. Summary.......... 3
2.2. Sampling design 3
2.3. Description of Sampling procedures 4
2.4. Sampling weights 5
2.5. Sampling weights 6

3. In house-validation of serological tests for the determination of antibodies against SARS-CoV-2.......... 7

3.1. Context 7
3.2. Study population.......... 8
3.3. Biological samples collection 8
3.4. Validation results 9
3.5. Comparison of tests 12
3.6. Processing costs 13
3.7. Conclusions..........13

4. Selection bias quantification.......... 14

Supplementary results **.......... 16**

1. Contextualization of seroprevalence in Mexico, 2020 16

2. COVID-19 related symptoms.......... 20

Supplementary references **.......... 21**

Supplementary methods

1. Study population included in Ensanut COVID 2020

Supplementary table 1. Description of the study population. Mexico 2020

	Sample size	Population in million	% (95% CI)
National	9,463	125.0	
Age (years)			
1-19	1,891	41.2	33.0 (31.4, 34.6)
20-39	2,954	38.7	30.9 (29.8, 32.1)
40-59	2,831	29.1	23.3 (22.3, 24.3)
60 and older	1,787	16.0	12.8 (12.0, 13.6)
Sex			
Male	3,976	60.9	48.8 (47.4, 50.1)
Female	5,487	64.0	51.2 (49.9, 52.6)
Education			
Elementary school or less	3,396	46.0	37.7 (36.1, 39.4)
Middle school	2,486	30.5	25.0 (23.7, 26.4)
High school	1,813	24.2	19.8 (18.7, 21.0)
Graduate	1,679	21.3	17.5 (16.1, 18.9)
Employment status[1]			
Unemployed	2,837	29.1	30.5 (29.2, 31.8)
Student	602	9.7	10.2 (9.2, 11.2)
Retired	457	4.1	4.3 (3.8, 4.9)
Formal worker[2]	1,526	19.3	20.2 (18.8, 21.7)
Informal worker	2,782	33.3	34.8 (33.2, 36.5)
Socioeconomic level			
Low	3,215	41.8	33.4 (31.0, 36.0)
Medium	3,130	40.6	32.5 (30.6, 34.5)
High	3,118	42.6	34.1 (31.9, 36.3)
Urbanization			
Rural	2,276	26.7	21.4 (19.3, 23.6)
Urban	2,899	37.5	30.0 (27.6, 32.5)
Metropolitan	4,289	60.8	48.6 (46.1, 51.2)

[1] Only for individuals with 15 years of age and more.

[2] Worker with access to social security services or private medical insurance.

2. External validity of ENSANUT COVID-19, 2020

2.1. Summary

ENSANUT COVID-19 is a probabilistic survey designed to achieve two goals: a) To study the impact of SARS-COV-2 on the health and nutrition of the Mexican population, and b) to estimate the trends of the main chronic diseases (diabetes, hypertension, and obesity). Probabilistic surveys are exercises of statistical inference; i.e., they try to make inferences from a sample to the population. Statistical inferences from a survey can be expressed through confidence intervals; the validity of confidence intervals can be supported in two ways. First, if a survey is probabilistic and measurements have no error, the intervals with 95% confidence for a parameter θ will contain the parameter 95% of the time. We will describe the sampling procedure to show that ENSANUT COVID-19 is a probabilistic survey, and support the validity of the confidence intervals when measurements have no error. Second, we will verify that estimators of ENSANUT COVID-19 for parameters that change slowly over time are similar to estimators of other surveys.

2.2. Sampling design

The usual way to make a probabilistic survey is to define a sampling frame, allocate probabilities of selection, and select a sample. The first step of ENSANUT COVID-19 is the construction of a sampling frame.

2.2.1 Sampling frame of Primary sampling units (PSU)

The sampling frame of PSU was a list of Basic Geostatistical Areas (AGEBs) built by the National Institute of Geography and Statistics (INEGI). In urban localities (2500 and more inhabitants), where usually a locality has more than one AGEB, the AGEBs of the 2010 Census was used as PSU. In contrast, the AGEB´s of the 2005 Population and Housing Count were used as PSU for the rural localities (1 to 2,499 inhabitants) because rural AGEB´s of the 2010 Census are not publicly available. AGEBs of the 2005 PCH were updated as follows: the new localities in the 2010 Census were added to rural AGEB´s and towns that disappeared in the 2010 Census were removed from the rural AGEBs.

2.2.2 Sampling frame of Secondary Sampling Units (SSU)

We used the list of urban blocks provided by INEGI for the public as the sampling frame of SSU for urban AGEBs. We used the list of rural localities as the sampling frame of SSU for rural AGEB.

2.2.3 Sampling frame of tertiary Sampling Units (SSU)

The National Institute of Public Health (INSP) constructed sampling frames for the SSU selected for the ENSANUT COVID-19 survey. In urban blocks, INSP made a list of households, and in rural localities, INSP made a list of clusters of households. The INSP team (INSP-cartography) that constructed the sampling frame of TSU was unrelated and independent of the household interviewers.

2.2.4 Sampling frame of individuals in households

Household interviewers made a list of all individuals in the households.

2.3. Description of Sampling procedures

2.3.1 Domains of study

Sampling size was set to make inferences for 9 regions of Mexico. Regions were defined as set of contiguous entities. The resulting regions were: Pacific-North (Baja California, Baja California Sur, Nayarit, Sinaloa, Sonora), Frontera (Chihuahua, Coahuila, Nuevo León, Tamaulipas), Pacific-Central (Colima, Jalisco, Michoacán), Central-North (Aguascalientes, Durango, Guanajuato, Querétaro, San Luís Potosí, Zacatecas), Centro (Aguascalientes, Durango, Guanajuato, Querétaro, San Luís Potosí, Zacatecas), Mexico City, State of Mexico, Pacific-South (Guerrero, Morelos, Oaxaca, Puebla) and Peninsula (Campeche, Chiapas, Quintana Roo, Tabasco, Yucatán). The sample size of complete households' interviews by region was: Pacific-North (1,035), Frontera (1,073), Pacific-Central (1,061), Central-North (1,768), Centro (1,033), Mexico City (1,163), State of Mexico (967), Pacific-South (1,084) and Peninsula (1,032).

2.3.2 Selection of primary sampling units

The primary sampling units (PSUs) were classified into three strata based on the size of the locality: rural (1 to 2,499 inhabitants), urban (2,500 to 99,999 inhabitants), and metropolitan (100,000 and more inhabitants). PSUs were selected with probability proportional to their population, and the sample size was allocated proportionally to the population cells of the contingency table defined by the cross-classification of entities and size of localities. The number of PSUs by region was: Pacific-North (42), Frontera (42), Pacific-Central (42), Central-North (67), Centro (42), Mexico City (64), State of Mexico (46), Pacific-South (41) and Peninsula (41).

2.3.3 Selection of secondary sampling units

The selection scheme depended on the type of stratum. In the PSUs of the urban and metropolitan strata, 5 blocks were selected with probability proportional to the population of the block according to the Census. Then, in each selected block, a selection of 6 households was made using systematic sampling with a random start; selection of households was carried out in the field by INSP-Cartography using a computer. In the case

of rural PSUs, 2 localities were selected with probability proportional to their size (total population). Later, during the field visit of INSP-Cartography, clusters of approximately 50 households were made in each locality; right away, 1 cluster was selected within each locality through a simple random sampling (SRS), and 1 sub-cluster of approximately 15 households was selected within the selected cluster, again, trough SRS.

2.3.4 Selection of people inside the households

The selection of participants within the households consisted of two stages. In the first stage, all households of a dwelling were identified and a household questionnaire was applied to each household (in Mexico more than one family or household could live in the same dwelling). The household questionnaire listed all the inhabitants and was stratified into six age groups. Supplementary table 2 specifies the sampling fraction for the age groups. ENSANUT COVID-19 selected a sample of health service users who received medical care in the last three months.

Supplementary table 2. Sampling fraction for individuals in the household

Group	Fraction of selection
Preschool Children from 0 to 4 years old	All
School Children 5 to 9 years old	One per household
Adolescents 10-19 years old	One per household
Adult 20-34 years old	One per household
Adult 35-49 years old	One per household
50 years old and over	One per household
Health service user	Up to 2 in 50% of the households

2.4. Sampling weights

ENSANUT COVID 19 selected individuals with a known probability, which was used to calculate the sampling weights. Sampling weights of ENSANUT COVID-19 were calculated on the basis of: a) probabilities of selection, b) response rates, and c) result of Census on the total number of individuals of Mexico. We expect that ENSANUT COVID 19 will produce unbiased estimators because weights are derived from probabilities of selection, and ENSANUT COVID 19 estimators resulted congenial to estimators of previous surveys, as is exemplified next.

2.5. Sampling weights

We compare ENSANUT-COVID19 estimators against external sources. We will present only three items for validation: the age pyramid, the prevalence of food insecurity, and the prevalence of diabetes. Supplementary figure 1 compares the age pyramid of the ENSANUT-COVID19 and the results of the Census 2020. Differences greater than 1% were not observed. Furthermore, ENSANUT-COVID-19 and the Census practically coincide in the percentage of men in households: 48% (ENSANUT-COVID19) and 49% (CENSUS).

Supplementary figure 1. Comparison of the age pyramids of the household population of ENSANUT-COV19 and the Census 2020 (N=36,024)

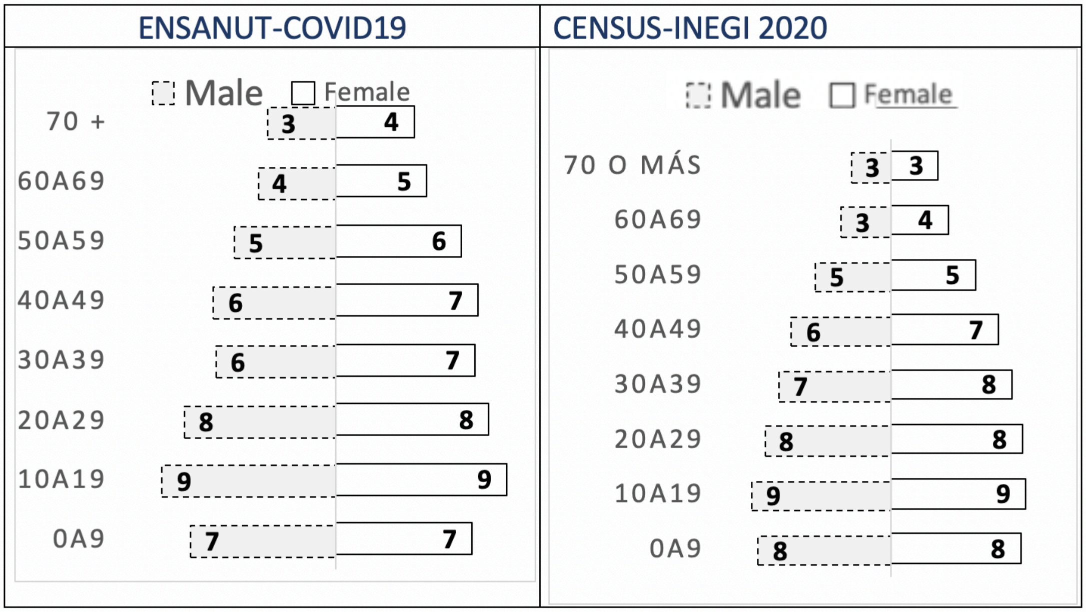

The numbers are percentages of each age group and sex from the total population.

Supplementary table 3 compares the prevalence of food insecurity between ENSANUT-COVID19 and ENSANUT 2018-2019. No statistically significant differences are observed. The food insecurity studied was defined as the prevalence of households lacking food due to a shortage of resources sometime in the past three months. Supplementary table 4 compares the prevalence of diabetes and shows no statistical difference between Ensanut 2018 and 2020.

Supplementary table 3. Percentage of households lacking food due to a shortage of resources in the past three months (N=10,206)

Locality size	ENSANUT 2020	ENSANUT 2018-2019
Rural	21.8% (19.2,24.8)	20.6% (19.3,21.9)

Urban	18.7% (16.9,20.7)	16.2% (15.4,17.1)
Metropolitan	12.9% (11.7,14.2)	11.6% (11.0,12.2)
Total	16.5% (15.5,17.8)	15.0% (14.5,15.6)

Supplementary table 4. Prevalence of diabetes (diagnosed + undiagnosed) by age

Age group (years old)	ENSANUT 2020	ENSANUT 2018-2019
20-29	2.2% (1.3,1.8)	3.2% (1.7,6.2)
30-39	7.6% (5.4,10.7)	7.7% (6.2,9.5)
40-49	18.6% (14.3,23.8)	16.2% (13.8,18.8)
50-59	28.6% (23.0,35.0)	28.9% (25.7,32.3)
60-69	28.1% (22.3,34.8)	34.0% (29.9,38.3)
70+	29.5% (22.6,37.6)	29.5% (25.5,33.7)
Total	15.7% (13.9,17.6)	16.8% (15.6,18.1)

3. In house-validation of serological tests for the determination of antibodies against SARS-CoV-2

3.1. Context

The Health Secretary announced that from August 2020, the National Health and Nutrition Survey 2020 (Ensanut 2020 Covid-19) would be collected at national level and by 9 regions: North-Pacific, Border, Center-Pacific, Center-North, Center, CDMX, State of Mexico, South-Pacific, Peninsula. This survey aims to provide information on the family experience of the pandemic, the effects on income, food security, diet quality, access to health services, and to measure SARS-CoV-2 antibodies to estimate the percentage of the population that has been exposed to coronavirus, under the coordination of the National Institute of Public Health (INSP).

The Institute for Epidemiological Diagnosis and Reference (InDRE), together with the INSP, worked on the processing of serological samples to evaluate the presence of specific antibodies against the SARS-CoV-2 virus. It is important to note that commercially available kits must be evaluated regarding sensitivity, specificity, positive and negative predictive values (PPV, NPV), and ROC curve prior to be used in studies for serological diagnosis. Here,

we report the results of an evaluation of three commercial tests aimed at detecting IgG antibodies against SARS-Cov-2.

3.2. Study population

We used a convenience sample of 326 people suspected of COVID-19 who attended the Family Medicine Unit (UMF) number 198 of the Mexican Social Security Institute (IMSS) between May and October 2020, in the municipality of Coacalco, State of Mexico. 155 samples had an initial follow-up, and the rest of the sample attended 30 days after the onset of symptoms for monitoring follow-up (171). All participants had a positive PCR result, but only 87 participants were able to retrieve the "*threshold cycle*". For the estimation of true negative values, 210 samples from the ENSANUT 2018 were used. The total sample of the study is 536 individuals.

Three commercial tests were used for the following evaluation. The first two were based on the chemiluminescence principle to detect antibodies directly against the nucleocapsid (N) of the virus and the third was an indirect ELISA directed at protein S. 326 positive and 210 negative samples were processed by the three tests:

- ROCHE's Elecsys Anti-SARS-CoV-2 (Sensitivity 100%, CI 88-100 and specificity 99.81%, CI [99.65-99.91, data reported by the manufacturer)
- ABBOTT SARS-CoV-2 IgG (Sensitivity 100%, CI 95.89-100 and specificity 99.60%, CI 98.98-100, data reported by the manufacturer).
- Elisa Anti SARS-CoV-2 (IgG) from the company EUROIMMUN (Sensitivity 94% and specificity 99.6%, the manufacturer does not report confidence intervals).

3.3. Biological samples collection

The medical staff of the UMF 198 was in charge of collecting the pharyngeal and nasopharyngeal exudates to detect SARS-CoV-2 using the reverse transcription technique coupled to the real-time polymerase chain reaction (rRT-PCR), and extracting blood samples to obtain serum. Pharyngeal and nasopharyngeal swab samples were processed by the IMSS and serum samples were sent to the InDRE SeroSurvey Laboratory for processing.

rRT-PCRs were obtained during the acute phase of infection (0-7 days). Blood samples were collected from patients with an initial diagnosis by PCR and patients recovered from infection at least 22 days after the onset of COVID-19 symptoms. The sera were processed by three different commercial kits for the determination of IgG class antibodies against SARS-CoV-2. Negative controls were provided by the INSP's ENSANUT-2018 biobank. 210

samples were randomly selected from the 32 states in Mexico, including areas where malaria is endemic, to consider the possibility of cross-reactivity. These samples do not have an rRT-PCR result, however, from an epidemiological moment prior to the emergence caused by SARS-CoV-2, they were assumed to be negative for infection.

3.4. Validation results

We evaluated the following parameters of the three different kits for serological analyses: sensitivity, specificity, positive predictive values, negative predictive values, the ROC curve, and the area under the curve.

ROCHE "Elecsys Anti-SARS-CoV-2"

Supplementary table 5. Contingency chart for the Elecsys Anti-SARS-CoV-2 assay

		Real-time RT-PCR		
		Positive	Negative	Total
ROCHE	Positive	300	1	301
	Negative	26	209	235
	nt	326	210	536

Supplementary table 6. Sensitivity and specificity for Elecsys Anti-SARS-CoV-2

Parameters	Value	Confidence intervals
Sensitivity	92.02%	88.57, 94.50
Specificity	99.52%	97.35, 99.92
PPV	99.97%	98.14, 99.94
NPV	88.90%	84.28, 92.34

From the 210 negative pre-pandemic samples, the ROCHE Elecsys Anti-SARS-CoV-2 kit had a false positive and 26 false negatives of the 326 positive samples tested (Supplementary table A1). This information should be interpreted with caution, as not all positive PCRs may have generated antibodies to SARS-CoV-2. The area under the curve was 97.7 with a confidence interval of 96.3 to 99.0%, statistical power of 1, and a significance level of 0.05. In Supplementary figure 2, the area shaded in blue represents the confidence intervals.

Supplementary figure 2. ROC curve of the Roche "Elecsys Anti-SARS-CoV-2" test

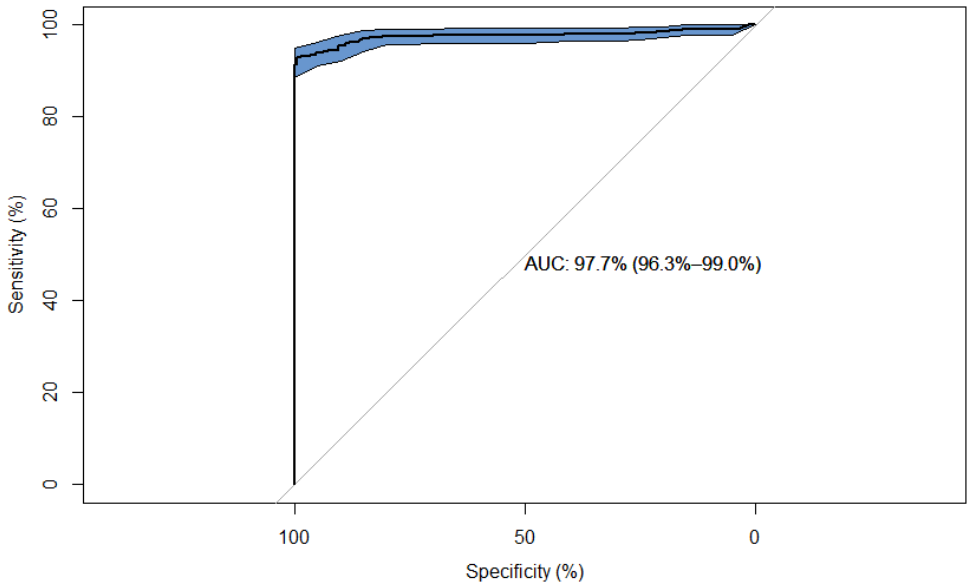

Error bands represent 95% confidence interval.

<u>ABBOTT SARS-CoV-2 IgG</u>

Supplementary table 7. Contingency chart for ABBOTT SARS-CoV-2 IgG assay

		REAL-TIME RT-PCR		
		Positive	Negative	Total
Abbott	Positive	301	6	307
	Negative	25	204	229
	Total	326	210	536

Supplementary table 8. Sensitivity and specificity for ABBOTT SARS-CoV-2 IgG

Parameters	Value	Confidence intervals
Sensitivity	92.00%	88.92 to 94.75
Specificity	97.14%	93.91, 98.68
PPV	98.00%	95.80, 99.10
NPV	88.91%	84.38, 92.50

From 210 negative samples, the ABBOTT SARS-CoV-2 IgG test, we observed six false positives and of the 326 positive samples, we observed 25 false negatives. The area under the curve for the ABBOTT test was 94.95% with a confidence interval of 92.9 to 97.0%, a

statistical power of 1, and a significance of 0.05 (Supplementary figure 3). For the results with available data of the cycle threshold (CT) values (n = 84), the results of the serological tests were adjusted by grouping the values into three age groups 0-20, 21-30 and 31-40. No significant differences were found between the groups.

Supplementary figure 3. ROC curve of the ABBOTT "SARS-CoV-2 IgG" test

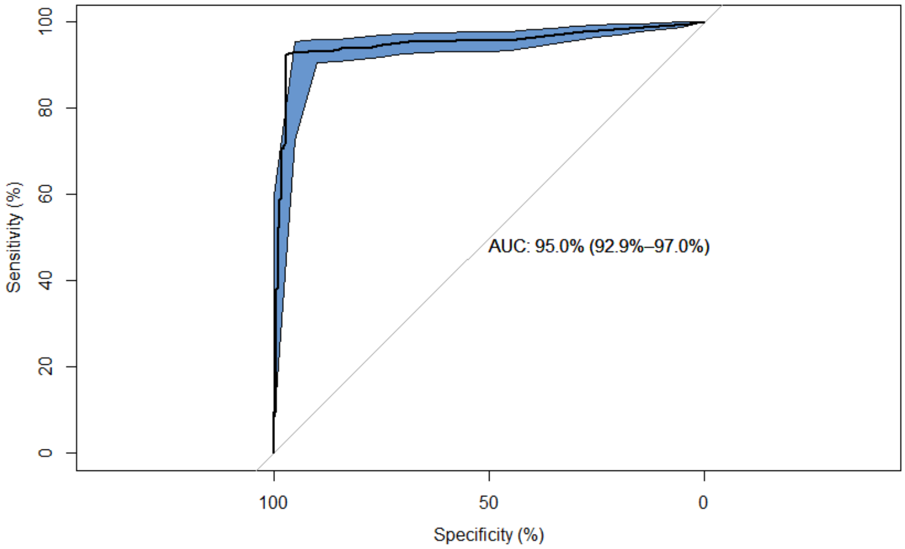

Error bands represent 95% confidence interval.

<u>EUROIMMUN Laboratory ELISA Anti SARS-CoV-2 (IgG)</u>

Supplementary table 9. Contingency chart for the EUROIMMUN Anti SARS-CoV-2 (IgG) ELISA assay.

		Real-time RT-PCR		
		Positive	Negative	Total
EUROIMMUN	Positive	299	4	303
	Negative	27	204	231
	Total	326	208	534

Supplementary table 10. Sensitivity and specificity for EUROIMMUN Anti SARS-CoV-2 (IgG) Elisa

Parameters	Value	Confidence intervals
Sensitivity	91.72%	88.22, 94.25
Specificity	98.1%	95.16, 99.25
PPV	98.7%	96.66, 99.49
NPV	88.3%	83.53, 91.84

From the 210 negative samples, we observed 4 false positives and 2 undetermined results, from the 326 positive samples, we observed 27 false negatives (Supplementary table 9). The area under the curve for the EUROIMMUN test was 96.2% with a confidence interval of 94.6 to 97.9%, statistical power of 1, and a significance of 0.05 (Supplementary figure 4).

Supplementary figure 4. ROC curve of the EUROIMMUN "Elisa Anti SARS-CoV-2 (IgG)" test

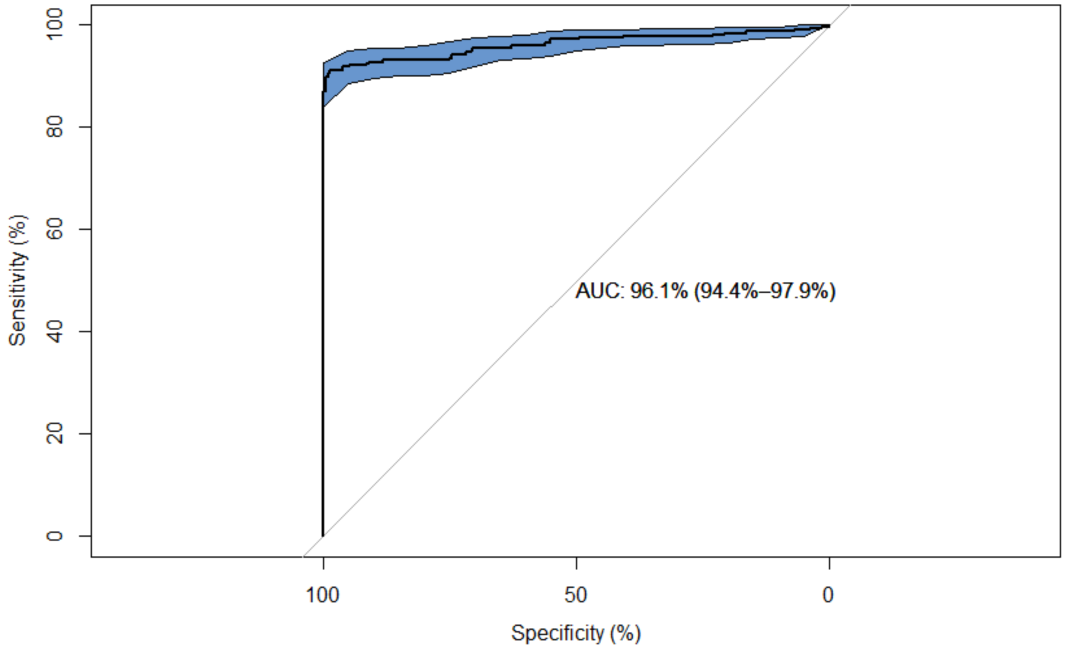

Error bands represent 95% confidence interval.

3.5. Comparison of tests

A comparison was made between the ROCHE laboratory test and the tests of the EUROIMMUNE and ABBOTT laboratories to evaluate possible significant differences (Supplementary figure 5). No significant differences were observed between EUROIMMUNE and ROCHE (p=0.110). However, a significant difference between the ROCHE and ABBOTT laboratory tests was found (p=0.009).

Supplementary figure 5. Test performance between A) ROCHE and EUROIMMUNE and B) ROCHE and ABBOTT

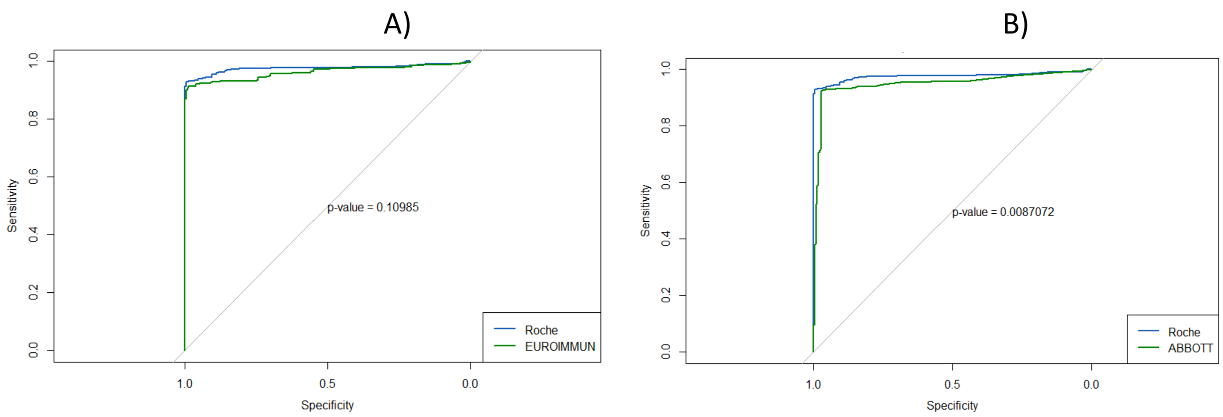

For the results with available data of the *CT* values (n = 84), the results of the ROCHE test values were adjusted by grouping them into three age groups "0-20, 21-30 and 31-40". No significant differences were found between the CT-adjusted age groups and the results of the sensitivity and specificity tests for any of the three tests.

3.6. Processing costs

The unit price of the Elecsys Anti SARS-CoV-2 test from the ROCHE laboratory was $48.16 MXN. Each kit can process 200 tests, so the total cost of each kit was $9,632.27 MXN. The cost per test for the "SARS-CoV-2 IgG" assay from the ABBOTT laboratory was $259.89 MXN, each kit contains 100 tests and the total cost per kit was $25,989.02 MXN. Finally, the "Elisa Anti SARS-CoV-2 (IgG)" test from the EUROIMMUN laboratory has a unit cost of $218.59 MXN, each kit can process 96 tests so the cost per kit is $20,984.40. The costs of all tests consider VAT included. Among the three tests, the ROCHE laboratory test had the lowest cost.

3.7. Conclusions

The three tests evaluated show adequate performance in detecting positive cases (sensitivity from 91.4 to 92%) and discriminating between true negatives and false positives (specificity from 97 to 99%). However, among the three tests, the Elecsys Anti-SARS-CoV-2 test from the ROCHE laboratory obtained the best score in terms of sensitivity and specificity (92% sensitivity and 99.4% specificity) and the IgG test of SARS-CoV-2 from ABBOTT Company performed worse (sensitivity of 91.4 and specificity of 97.14). The most important variation between the tests are the false positives evaluated in the ENSANUT-

2018 samples, before the appearance of SARS-CoV-2. ROCHE's laboratory test produced only one false positive, ABBOTT six, and EUROIMMUN four false positives and two indeterminate results. To estimate false negatives, the information should be interpreted with caution, as not all people infected with SARS-CoV-2 will generate antibodies.

Whereas ROCHE and ABBOTT's lab tests target the nucleocapsid, the EUROIMMUN "Elisa Anti SARS-CoV-2 (IgG)" test targets protein S, so its results should be more specific for the SARS-CoV-2. However, the unit cost of the EUROIMMUN laboratory tests is 4.5 times higher than the unit cost of the ROCHE laboratory tests. In this sense, although all the tests respond adequately in their sensitivity and specificity values, the ROCHE test has a better cost-benefit performance. Therefore, this evaluation recommends the use of the "Elecsys Anti-SARS-CoV-2" test from the ROCHE laboratory for the processing and detection of anti-SARS-CoV-2 IgG antibodies in the samples obtained through the ENSANUT survey. COVID 2020.

4. Selection bias quantification

We evaluated the possibility of selection bias considering low response rate in the serologic sample. First, we selected variables associated with seroprevalence that could potentially affect the probability of accepting to participate in the serologic subsample: age, sex, region, education, employment, contact with a suspected case of COVID, having a respiratory disease, having experienced COVID-19 related symptoms. Then, we compared the distributions of those variables between the household questionnaire (36,024 subjects) and the serologic subsample (9,464 subjects). In that comparison, we considered that the household sample is a more representative sample of the population for two reasons: the sample size, and the proportion of people who agreed to participate (serologic vs household sample: 44% vs 73%).[1] We found that the serologic subsample had a lower proportion of students, and a higher proportion of people reporting contact with a suspected case, having a respiratory disease or symptoms, and reporting difficulty breathing (Supplementary table 11). We used raking, a sampling balance method, to replicate the distribution of the key variables from the household sample into the serologic subsample. Our first approach was to use the least key variables, because the variables could be correlated and using too many variables increases the complexity and reduces the efficiency. Using the household distribution of “symptoms” by age and region to adjust the sample distribution, we found that all the distribution of the serologic sample variables--except students--, now fall within the 95% CI of the distribution of the household sample (see Supplementary table 11). For example, difficulty breathing was reported by 2.4% (95%CI 2.2,2.6) of the individuals in the household sample, but among

3.4% (95%CI 3.0,3.9) in the serologic subsample. After the correction, the estimate in the serologic subsample matched the household sample with 2.5% (95%CI 2.2,2.8).

Supplementary table 11. Distribution of socioeconomic and Covid-19 related characteristics between the household sample, household sample and serologic subsample before and after adjusting for selection bias.

	Household sample n=36,024			Serologic sample n=9,464				
				Before raking			After raking	
	N (million)	%	IC95%	N (million)	%	IC95%	%	IC95%
Age group								
1-9	19.2	15.4	[14.8,16.0]	19.3	15.4	[14.1,16.9]	15.4	[14.0,16.8]
10-19	21.9	17.5	[16.9,18.1]	21.9	17.6	[16.6,18.6]	17.6	[16.6,18.6]
20-29	21.1	16.9	[16.4,17.4]	22.7	18.1	[17.2,19.1]	18.0	[17.2,19.0]
30-39	17.2	13.7	[13.3,14.2]	16.0	12.8	[12.0,13.7]	12.6	[11.8,13.5]
40-49	17.4	13.9	[13.4,14.4]	17.7	14.2	[13.4,15.0]	14.2	[13.5,15.0]
50-59	11.9	9.5	[9.1,9.9]	11.4	9.1	[8.5,9.8]	9.2	[8.5,9.8]
60-69	8.9	7.2	[6.8,7.6]	8.6	6.9	[6.4,7.5]	7.0	[6.5,7.6]
70-79	5.1	4.1	[3.8,4.4]	5.5	4.4	[4.0,4.9]	4.5	[4.0,4.9]
80+	2.3	1.8	[1.7,2.0]	1.9	1.5	[1.3,1.8]	1.5	[1.3,1.8]
Sex								
Men	61.9	48.8	[48.4,49.2]	60.9	48.8	[47.4,50.1]	48.7	[47.4,50.1]
Women	65.0	51.2	[50.8,51.6]	64.0	51.2	[49.9,52.6]	51.3	[49.9,52.6]
Regions								
Pacific-North	11.8	9.3	[8.1,10.8]	11.7	9.4	[7.9,11.0]	9.4	[7.9,11.0]
Border	16.3	12.9	[11.2,14.7]	16.1	12.9	[11.2,14.7]	12.9	[11.2,14.7]
Pacific-Center	13.9	11.0	[10.1,11.9]	13.7	11.0	[9.9,12.1]	11.0	[9.9,12.2]
Center-North	16.3	12.9	[11.4,14.5]	16.1	12.9	[11.1,14.8]	12.9	[11.1,14.9]
Center	12.6	9.9	[9.1,10.8]	12.4	9.9	[9.0,10.9]	9.9	[9.0,10.9]
Mexico City	9.3	7.3	[6.8,7.9]	9.2	7.4	[6.7,8.0]	7.4	[6.7,8.1]
State of Mexico	17.1	13.5	[12.6,14.5]	16.9	13.5	[12.3,14.7]	13.5	[12.3,14.7]
Pacific South	16.3	12.9	[11.8,14.0]	16.0	12.8	[11.7,14.1]	12.8	[11.7,14.0]
Peninsula	13.1	10.4	[9.0,11.9]	12.9	10.3	[8.8,12.2]	10.4	[8.8,12.2]
Education								
Elementary or less	45.3	37.4	[36.4,38.5]	46.0	37.7	[36.1,39.4]	38.0	[36.4,39.7]
Middle school	31.2	25.7	[24.9,26.5]	30.5	25	[23.7,26.4]	25.1	[23.8,26.4]
High school	23.6	19.5	[18.8,20.2]	24.2	19.8	[18.7,21.0]	19.6	[18.5,20.7]
Graduate	21.0	17.4	[16.3,18.5]	21.3	17.5	[16.1,18.9]	17.3	[15.9,18.8]
Employment status								
Unemployed	29.6	29.5	[28.8,30.3]	29.1	30.5	[29.2,31.8]	30.8	[29.5,32.1]
Student	13.8	13.7	[13.2,14.3]	9.7	10.2	[9.2,11.2]	10.2	[9.2,11.2]

Retired	4.2	4.2	[3.8,4.6]	4.1	4.3	[3.8,4.9]	4.4	[3.9,5.0]
Formal worker	19.6	19.6	[18.8,20.4]	19.3	20.2	[18.8,21.7]	20.0	[18.6,21.4]
Informal worker	33.1	33	[32.1,33.9]	33.3	34.8	[33.2,36.5]	34.6	[33.1,36.2]
Contact with a suspected case of COVID								
No	117.8	92.8	[92.2,93.5]	113.0	90.4	[89.4,91.4]	91.5	[90.5,92.4]
Yes	9.1	7.2	[6.5,7.8]	11.9	9.6	[8.6,10.6]	8.5	[7.6,9.5]
Reporting a respiratory disease								
Yes	13.9	11	[10.4,11.6]	17.6	14.1	[13.1,15.2]	10.9	[10.1,11.8]
No	113.0	89	[88.4,89.6]	107.3	85.9	[84.8,86.9]	89.1	[88.2,89.9]
Symptoms								
Asymptomatic	109.6	86.4	[85.7,87.0]	102.3	81.9	[80.6,83.1]	86.3	[85.3,87.3]
Symptomatic	9.1	7.2	[6.7,7.7]	12.6	10.1	[9.2,11.1]	7.2	[6.6,7.9]
Pauci-symptomatic	8.2	6.5	[6.1,6.8]	10.1	8.1	[7.3,8.8]	6.5	[5.9,7.1]
Reporting difficulty breathing								
No	123.8	97.6	[97.4,97.8]	120.7	96.6	[96.1,97.0]	97.5	[97.2,97.8]
Yes	3.1	2.4	[2.2,2.6]	4.3	3.4	[3.0,3.9]	2.5	[2.2,2.8]

Supplementary results

1. Contextualization of seroprevalence in Mexico, 2020

Supplementary figure 6. Daily confirmed cases by RT-PCR by region.

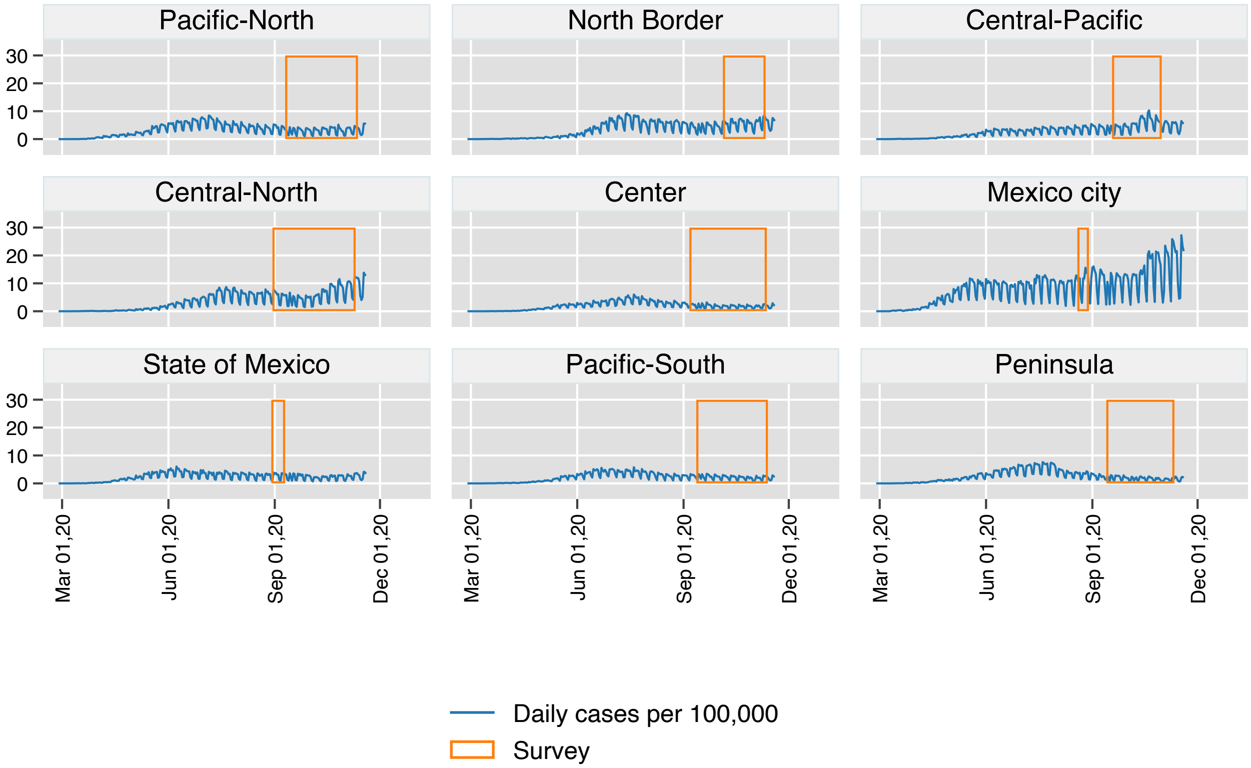

The y axis shows the rate per 100,000 inhabitants.

For most regions, the Ensanut collection period occurred in the plateau after the first epidemic wave (Supplementary figure 6). The only exception is Central North and Central Pacific, where the end of the collection period occurred at the beginning of the second wave. Peninsula presented the highest seroprevalence in Ensanut (42.9%), which was not confirmed by surveillance data in which new confirmed cases did not exceed 9 cases per 100,000 inhabitants, even in the first wave peak.

Supplementary table 12. Cumulative number of cases per 100,000 inhabitants (SISVER) and number of tests in each region at the midpoint of the survey

	Incident cases	Seropositivity	Ratio Cases: Seroprevalence	Tests (mid-point for each region)	Tests (mid-point survey: Sept 30th)
Pacific-North	712	31.0	44	1346	1616
North Border	763	21.0	28	1660	1891
Central-Pacific	495	19.4	39	1111	1452

Central-North	627	19.1	30	1482	2111
Center	440	25.5	58	789	939
Mexico City	1067	19.6	18	2692	5931
State of Mexico	431	23.5	55	969	1388
Pacific-South	485	24.3	50	898	1045
Peninsula	586	42.9	73	1177	1377
National	600	24.9	41	1289	1802

Supplementary figure 7. Seroprevalence of SARS-CoV.2 in Mexico and other countries from April to November 2020.

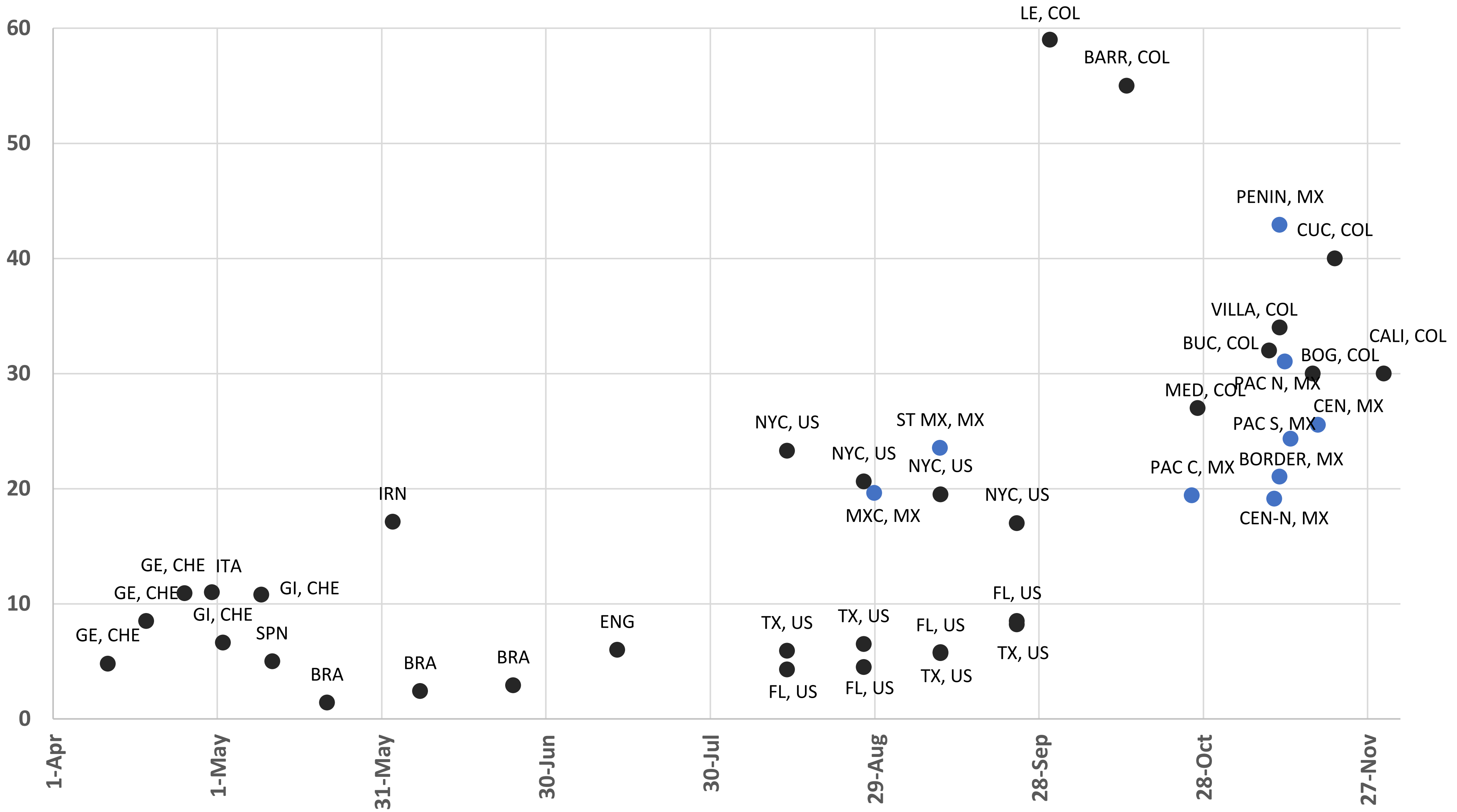

In black other countries and in blue Mexico. GE, CHE: Geneva, Switzerland, ITA: Liguria and Lombardia (Italy), SPN: Spain, BRA: Brazil (27 federative units), IRN: Iran, ENG: England, TX, US: Texas, FL, US: Florida, NYS, US: New York, MXC, MX: Mexico City, ST MX: State of Mexico. PAC C, MX: Pacific Center Mexico, CEN-N, MX, Center north Mexico, PAC S, MX: Pacific South Mexico, CEN, MX: Center Mexico, PAC N, MX: Pacific north, Penin, Mx: Peninsula Mexico. MED, COL: Medellin Colombia, BARR, COL: Barranquilla Colombia, LE, COL: Leticia Colombia, BUC, COL: Bucaramanga, Colombia, VILLA, COL: Villavicencio, Colombia, BOG, COL: Bogotá, Colombia, CUC, COL: Cúcuta, Colombia. [2–9]

2. COVID-19 related symptoms

Supplementary figure 8. Symptoms prevalence (%) of COVID-19 related symptoms by serostatus

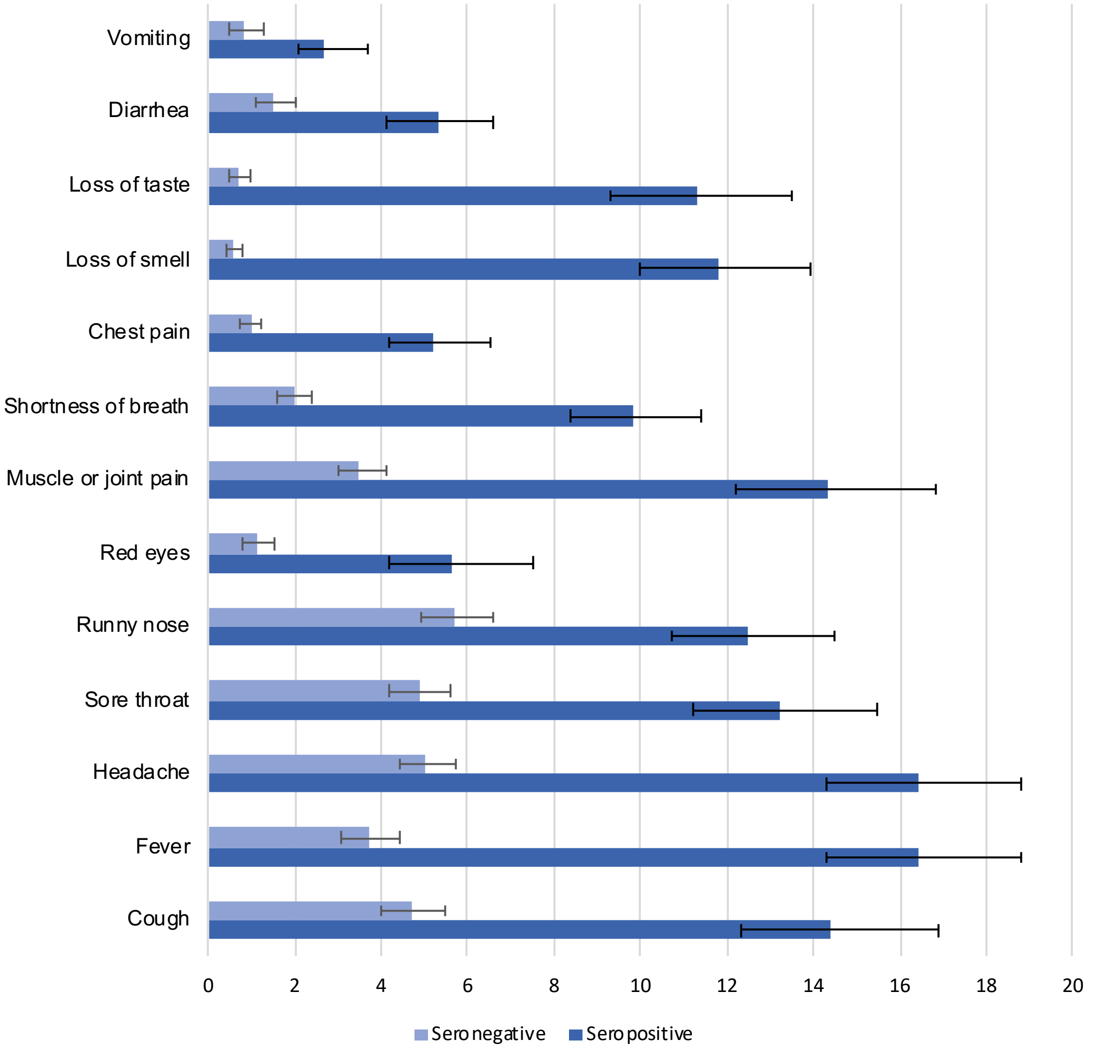

The sample size of independent individuals is n=9,464. Error bars represent 95% confidence intervals. Muscle and joint pain were asked in the same question.

Supplementary references

1. Romero-Martínez, M. *et al.* National Health and Nutrition Survey 2020 Covid-19 methodology. *Salud Publica Mex* **63**, 444–451 (2021).
2. Poustchi, H. *et al.* SARS-CoV-2 antibody seroprevalence in the general population and high-risk occupational groups across 18 cities in Iran: a population-based cross-sectional study. *Lancet Infect. Dis.* **2**, 1–9 (2021).
3. Pollán, M. *et al.* Prevalence of SARS-CoV-2 in Spain (ENE-COVID): a nationwide, population-based seroepidemiological study. *Lancet* **396**, 535–544 (2020).
4. Stringhini, S. *et al.* Seroprevalence of anti-SARS-CoV-2 IgG antibodies in Geneva, Switzerland (SEROCoV-POP): a population-based study. *Lancet* **396**, 313–319 (2020).
5. Horta, B. L. *et al.* Prevalence of antibodies against SARS-CoV-2 according to socioeconomic and ethnic status in a nationwide Brazilian survey. *Rev. Panam. Salud Publica/Pan Am. J. Public Heal.* **40**, 1–7 (2020).
6. Vena, A. *et al.* Prevalence of Antibodies to SARS-CoV-2 in Italian Adults and Associated Risk Factors. *J. Clin. Med.* **9**, 2780 (2020).
7. Instituto Nacional de Salud. Estudio Nacional: Seroprevalencia. Durante la epidemia en Colombia. (2021). Available at: https://www.ins.gov.co/estudio-nacional-de-seroprevalencia/reporte.html#curso. (Accessed: 16th March 2021)
8. Bajema, K. L. *et al.* Estimated SARS-CoV-2 Seroprevalence in the US as of September 2020. *JAMA Intern. Med.* **30329**, 1–11 (2020).
9. Ward, H. *et al.* SARS-CoV-2 antibody prevalence in England following the first peak of the pandemic. *Nat. Commun.* **12**, 1–8 (2021).
