# Supplementary material for: Nationally representative SARS-CoV-2 antibody prevalence estimates after the first epidemic wave in Mexico
Source: Nat Commun. 2022 Feb 1;13:589. doi: 10.1038/s41467-022-28232-9 (PMC8807586; doi:10.1038/s41467-022-28232-9)
Supplement: Supplementary file 2 — Reporting Summary [file 41467_2022_28232_MOESM2_ESM.pdf]

nature research

Corresponding author(s): Tonatiuh Barrientos Gutierrez

Last updated by author(s): Nov 30, 2021

Reporting Summary

Nature Research wishes to improve the reproducibility of the work that we publish. This form provides structure for consistency and transparency in reporting. For further information on Nature Research policies, see our Editorial Policies and the Editorial Policy Checklist.

Statistics

For all statistical analyses, confirm that the following items are present in the figure legend, table legend, main text, or Methods section.

n/a	Confirmed	
☐	☒	The exact sample size (*n*) for each experimental group/condition, given as a discrete number and unit of measurement
☐	☒	A statement on whether measurements were taken from distinct samples or whether the same sample was measured repeatedly
☐	☒	The statistical test(s) used AND whether they are one- or two-sided<br>*Only common tests should be described solely by name; describe more complex techniques in the Methods section.*
☐	☒	A description of all covariates tested
☐	☒	A description of any assumptions or corrections, such as tests of normality and adjustment for multiple comparisons
☐	☒	A full description of the statistical parameters including central tendency (e.g. means) or other basic estimates (e.g. regression coefficient) AND variation (e.g. standard deviation) or associated estimates of uncertainty (e.g. confidence intervals)
☐	☒	For null hypothesis testing, the test statistic (e.g. *F*, *t*, *r*) with confidence intervals, effect sizes, degrees of freedom and *P* value noted<br>*Give P values as exact values whenever suitable.*
☒	☐	For Bayesian analysis, information on the choice of priors and Markov chain Monte Carlo settings
☒	☐	For hierarchical and complex designs, identification of the appropriate level for tests and full reporting of outcomes
☐	☒	Estimates of effect sizes (e.g. Cohen's *d*, Pearson's *r*), indicating how they were calculated

Our web collection on statistics for biologists contains articles on many of the points above.

Software and code

Policy information about availability of computer code

Data collection	The information in the field was collected on tablets through a capture system developed in the CSPro language version 7.5.0, a publicly available software developed by the United States Census Bureau.
Data analysis	All analyses considered survey weights using the module “svy” from Stata 14.0 (College Station, TX). The data is publicly available in https://ensanut.insp.mx/encuestas/ensanutcontinua2020/descargas.php. The script for main analysis and sensitivity analysis, an excel file to adjust the estimation by in-house test performance and the dataset for sensitivity analysis are provided in GitHub. We linked it to the zenode with the reference: Code availability for the paper Seroprevalence in Mexico published in nature communication. DOI: 10.5281/zenodo.5745227

For manuscripts utilizing custom algorithms or software that are central to the research but not yet described in published literature, software must be made available to editors and reviewers. We strongly encourage code deposition in a community repository (e.g. GitHub). See the Nature Research guidelines for submitting code & software for further information.

Data

Policy information about availability of data

All manuscripts must include a data availability statement. This statement should provide the following information, where applicable:

- Accession codes, unique identifiers, or web links for publicly available datasets
- A list of figures that have associated raw data
- A description of any restrictions on data availability

Data is publicly available in https://ensanut.insp.mx/encuestas/ensanutcontinua2020/descargas.php; folio_int is the unique identifier and ponde_g20 is the weight variable to expand the results to the Mexican population.

Field-specific reporting

Please select the one below that is the best fit for your research. If you are not sure, read the appropriate sections before making your selection.

☐ Life sciences ☒ Behavioural & social sciences ☐ Ecological, evolutionary & environmental sciences

For a reference copy of the document with all sections, see nature.com/documents/nr-reporting-summary-flat.pdf

Behavioural & social sciences study design

All studies must disclose on these points even when the disclosure is negative.

Study description	Quantitative cross-sectional study.
Research sample	Research sample with 9,464 individuals from 1 to 99 years of age from Mexico, representing 124,950,278 individuals. The dataset with is is publicly available in https://ensanut.insp.mx/encuestas/ensanutcontinua2020/descargas.php
Sampling strategy	Used a multistage, probabilistic and stratified sampling to select 10,216 participating households. The sampling procedure was carried out in two stages: selection of households and individuals. From 35,632 eligible participants in the household survey, 21,707 individuals were randomly selected to provide a blood sample following a multistage and stratified selection strategy divided into six age groups: 1-4 years, 5-9 years, 10-19 years, 20-34 years, 35-49 years and 50+ years. This strategy allowed us to have 9,464 serum samples to measure seroprevalence and allows us to have representation at national and regional level.<br><br>The sample size was calculated using the formula:<br>m=Z^2 (p(1-p))/(δ) Deff<br>where m = Sample size in serum, p = Seroprevalence to estimate (5%, taken from the seroprevalence study in Spain); Z = Quantile 97.5% of a normal distribution (Z = 1.96), δ is the semi-amplitude (0.02) of the confidence interval and the design effect (Deff) is 2: number of units collected by a specific sampling procedure.
Data collection	The data collection was obtained by the ENSANUT team in the household, measures were taken to allow for the confidentiality of response regarding other household members. No experimental condition exists in this paper. The information in the field was collected on tablets through a capture system developed in the CSPro language version 7.5.0, a publicly available software developed by the United States Census Bureau
Timing	The survey was conducted between 18th August and 13th November 2020.
Data exclusions	From the total 9,640 blood samples collected, 176 samples were excluded because had insufficient sample to be analyzed or were lost.
Non-participation	44% response rate from eligible participants. The reason for dropout is explained in detail in figure 2 in the methods section: 13% could not be contacted, 40% declined and 2% rejected being punctured. We performed a sensitivity analysis to assess the potential impact of specific variables that could inform selection bias, considering a low response rate in the serologic subsample. Using the selection bias adjustment, we found small variations across regions; the overall pattern remained unchanged.
Randomization	Not applicable because this is not a expermental study.

Reporting for specific materials, systems and methods

We require information from authors about some types of materials, experimental systems and methods used in many studies. Here, indicate whether each material, system or method listed is relevant to your study. If you are not sure if a list item applies to your research, read the appropriate section before selecting a response.

Materials & experimental systems

n/a	Involved in the study	
☐	☒	Antibodies
☒	☐	Eukaryotic cell lines
☒	☐	Palaeontology and archaeology
☒	☐	Animals and other organisms
☐	☒	Human research participants
☒	☐	Clinical data
☒	☐	Dual use research of concern

Methods

n/a	Involved in the study	
☒	☐	ChIP-seq
☒	☐	Flow cytometry
☒	☐	MRI-based neuroimaging

Antibodies

Antibodies used	We used the Roche Elecsys Anti-SARS-CoV-2 pan-immunoglobulin immunoassay test to determine the immunoglobulin G against

Antibodies used	nucleocapsid protein. According to the manufacturer recommendations, samples were considered reactive using a threshold of ≥1.0 AU/ml.
Validation	We validated the test using pre-COVID-19 serum samples as controls and serum samples from people with confirmed COVID-19 by RT-PCR that were obtained at least 22 days after symptoms onset, when the sensitivity of the antibody tests is the highest.

Human research participants

Policy information about studies involving human research participants

Population characteristics	See above
Recruitment	From 35,632 eligible participants in the household survey, 21,707 individuals were randomly selected to provide a blood sample following a multistage and stratified selection strategy divided into six age groups: 1-4 years, 5-9 years, 10-19 years, 20-34 years, 35-49 years and 50+ years. A total of 9,640 blood samples were collected, for a 44% response rate from eligible participants. The reason for dropout is explained in detail in figure 2 in the methods section: 13% could not be contacted, 40% declined and 2% rejected being punctured. We performed a sensitivity analysis to assess the potential impact of specific variables that could inform selection bias, considering a low response rate in the serologic subsample. Using the selection bias adjustment, we found small variations across regions; the overall pattern remained unchanged.
Ethics oversight	All research procedures were approved by the ethics, research and biosafety boards from the National Institute of Public Health.

Note that full information on the approval of the study protocol must also be provided in the manuscript.
